# Police Matrons

**Published:** 1889-03-16

**Authors:** 


### POLICE MATRONS.

MISS LOUISA TWINING writes: As there appears to be some confusion as to the different classes of police and prison matrons in your recent article, you will perhaps allow me to explain the difference and distinction. In the first instance the present movement is in no way concerned with prison matrons or arrangements, for there appears to be no need for interference with that department. Women only are appointed to supervise women, and this makes the other arrangements a more remarkable contrast. Male warders are not allowed to enter the women's side of the prison, and they have no keys of it. The subject under discussion is therefore only concerning police-court and police-station management, and as few persons seem to understand the distinction, I may explain that the arrangements of the former alone were the subject of the recent inquiry and Commission ordered by Government. To the police courts persons are taken from the stations and from prisons to appear before the magistrates, and these have been fully described by the five Commissioners appointed to report upon them. For these alone matrons have been recommended and structural improvements ordered. Then there are the police stations, apart and wholly separate from the courts. To these persons are taken at once on being arrested by the police, and frequently detained all night, till discharged, or sent to the courts before the magistrates. These have not been included nor named in the recent Report, though the need of reform is as great, if not greater, as I explained in my recent letter to the *Times*. It is to this last object alone, therefore, that we are now urging public attention, in the hope that resident matrons may be appointed to all police stations as well as courts, instead of the at present non-resident female searchers.

